# A Method for Producing Transgenic Cells Using a Multi-Integrase System on a Human Artificial Chromosome Vector

**DOI:** 10.1371/journal.pone.0017267

**Published:** 2011-02-24

**Authors:** Shigeyuki Yamaguchi, Yasuhiro Kazuki, Yuji Nakayama, Eiji Nanba, Mitsuo Oshimura, Tetsuya Ohbayashi

**Affiliations:** 1 Department of Biomedical Science, Institute of Regenerative Medicine and Biofunction, Graduate School of Medical Sciences, Tottori University, Yonago, Japan; 2 Division of Laboratory Animal Science, Research Center for Bioscience and Technology, Tottori University, Yonago, Japan; 3 Chromosome Engineering Research Center, Tottori University, Yonago, Japan; 4 Division of Functional Genomics, Research Center for Bioscience and Technology, Tottori University, Yonago, Japan; City of Hope National Medical Center and Beckman Research Institute, United States of America

## Abstract

The production of cells capable of expressing gene(s) of interest is important for a variety of applications in biomedicine and biotechnology, including gene therapy and animal transgenesis. The ability to insert transgenes at a precise location in the genome, using site-specific recombinases such as Cre, FLP, and ΦC31, has major benefits for the efficiency of transgenesis. Recent work on integrases from ΦC31, R4, TP901-1 and Bxb1 phages demonstrated that these recombinases catalyze site-specific recombination in mammalian cells. In the present study, we examined the activities of integrases on site-specific recombination and gene expression in mammalian cells. We designed a human artificial chromosome (HAC) vector containing five recombination sites (ΦC31 *attP*, R4 *attP*, TP901-1 *attP*, Bxb1 *attP* and FRT; multi-integrase HAC vector) and *de novo* mammalian codon-optimized integrases. The multi-integrase HAC vector has several functions, including gene integration in a precise locus and avoiding genomic position effects; therefore, it was used as a platform to investigate integrase activities. Integrases carried out site-specific recombination at frequencies ranging from 39.3–96.8%. Additionally, we observed homogenous gene expression in 77.3–87.5% of colonies obtained using the multi-integrase HAC vector. This vector is also transferable to another cell line, and is capable of accepting genes of interest in this environment. These data suggest that integrases have high DNA recombination efficiencies in mammalian cells. The multi-integrase HAC vector enables us to produce transgene-expressing cells efficiently and create platform cell lines for gene expression.

## Introduction

Many methods are available to produce transgenic cells for the functional studies of genes, drug discovery and gene therapy. The most common method used to produce these cells relies on random integration of the gene after transfection of plasmid DNA or transduction with viruses. These methods are followed by antibiotic selection of a stable pool of cells and functional screening to identify individual clones that have the correct function(s). However, random integration into chromosomes is inefficient [1], and the expression levels of genes vary greatly due to positional effects and the number of copies inserted [2], [3], [4], [5]. As a result, the process of generating and selecting gene expression cells can be labor intensive and extremely time consuming. It is a widely held view that new gene expression technology for mammalian cells should optimally include targeting the gene to a transcriptional ‘hot spot’ in the genome [6]. Although homologous recombination for targeted integration is very specific, it suffers from exceedingly low frequencies [7].

To increase the speed and efficiency of generating transgenic cells, alternative technologies have been considered. The site-specific gene recombination systems, such as bacteriophage P1-derived Cre, yeast-derived FLP, and phage integrases typified by bacteriophage ΦC31-derived integrase, are example of these. These systems have been used widely for the targeted recombination of transgenes into the genome of mammalian cells [8]. Additionally, these site-specific recombinases can induce the deletion or inversion of DNA sequences leading to conditional gene inactivation or expression [9]. The most powerful tool for site-specific recombination *in vitro* [10], [11] and *in vivo* [12], [13] is Cre recombinase, which catalyzes reciprocal site-specific recombination between two loxP sites. A second site-specific recombinase, FLPe, based on FLP from *Saccharomyces cerevisiae*, has also been used in mammalian cells and recognizes distinct FRT sites [14]. FLPe is an improved and temperature stable version of the FLP recombinase. However, in assays with chromosomally located FRT sites, the efficiency of FLPe only exhibits 10% Cre recombination activity [15]. A third class of site-specific recombinases, the serine integrases, as typified by ΦC31 integrase, also displays activity in mammalian cells. Tyrosine integrases such as λ phage integrase are also used in mammalian cells [16], [17]. However, the recombination efficiency of tyrosine family integrases is lower than that of serine family integrases, and we therefore used serine integrases in this study [18].

The ΦC31 integrase was originally isolated from a *Streptomyces* phage [19], and the 605 amino acid ΦC31 integrase can perform recombination between *attP* and *attB* sites, which is different to Cre and FLPe in human cells [20]. Recombination between *attP* and *attB* sites generates hybrid *attL* or *attR* sites that are no longer substrates for the integrase in the absence of additional cofactors [20], [21]. Furthermore, ΦC31 integrase facilitates integration of *attB*-bearing plasmids at endogenous sequences with partial identity to *attP*. These are termed pseudo *attP* sites [22]. The ability of ΦC31 integrase to mediate transgene integration into native pseudo *attP* sites has been used in gene therapy experiments to produce therapeutically useful levels of Factor IX, correct human type VII collagen genes in human keratinocytes that contained mutants of this gene [18], and to produce dystrophin in mouse muscle-derived stem cells, human myoblasts and mouse muscle [23], [24]. In addition, ΦC31 integrase has been used in the construction and manipulation of transgenic animals [25], [26], [27]. Based on these favorable results with ΦC31 integrase, other serine integrases from phages such as R4, TP901-1 and Bxb1, have been evaluated in mammalian cells. The R4 integrase (469 amino acids) is a site-specific, unidirectional recombinase derived from the genome of phage R4 of *Streptomyces parvulus* [28], [29]. The TP901-1 integrase (485 amino acids) is encoded by phage TP901-1 of *Lactococcus lactis subsp* [30], [31]. The Bxb1 integrase (500 amino acids) is derived from mycobacteriophage Bxb1, which is a temperate phage of *Mycobacterium smegmatis* [32], [33]. Recent work on integrases from R4, TP901-1 and Bxb1 phages demonstrates that these enzymes mediate DNA recombination at heterotypic binding sequences known as *attB* and *attP* sites in mammalian cells [28], [30], [34]. One report suggests that ΦC31 integrase may have high recombination efficiency (87%), similar to Cre, in mediating recombination in cultured cells [15]. However, other integrases have demonstrated more limited success, and thus the broad utility of phage integrases as a tool remains to be established. Codon usage bias has been reported for many organisms, from viruses to eukaryotes [35]. If a gene contains codons that are rarely used in the host, its level of expression will not be maximal. Codon-optimization involves altering the rare codons in the target gene so that they more closely reflect the codon usage of the host, without modifying the amino acid sequence of the encoded protein. The ability of phage integrases, from phages ΦC31, R4, TP901-1 and Bxb1, to carry out efficient and precise recombination between their *attP* and *attB* sequences in mammalian cells will make them new and valuable genetic tools for the engineering of complex genomes.

Human artificial chromosomes (HACs) are highly promising gene delivery tools that possess several advantages over conventional gene delivery systems [36], [37]. We recently produced HAC vectors by deleting all of the endogenous genes on human chromosome 21 [38], [39]. Like native chromosomes, the HAC vector has the capacity to replicate and segregate autonomously without integration into the host genome. The genomic context of the host chromosome is disrupted by the transgene [40] and expression of the transgene is ruled by positional effects, due to random integration. The HAC vector can avoid effects on both the transgene and the host genome. Therefore, we considered that the HAC vector was an appropriate platform for measuring site-specific recombination activities of phage integrases in various mammalian cells. Additionally, the HAC vector was transferred from Chinese hamster ovary (CHO) cells to various other mouse and human cell lines by microcell-mediated chromosome transfer (MMCT) [38], [41], [42]. The CHO cell line, which is a capable microcell donor, has been used as an intermediate host of transferable chromosome fragments to other cell lines [43].

In this study, we designed a HAC-based multi-integrase system containing five recombination sites (ΦC31 *attP*, R4 *attP*, TP901-1 *attP*, Bxb1 *attP* and FRT) and integrases. We carried out the present study to examine the site-specific recombination activity and the actual efficacy of producing transgenic mammalian cells by these integrases.

## Results

### Construction of the multi-integrase HAC vector (MI-HAC vector) in Chinese hamster ovary (CHO) cells

**Figure 1:**
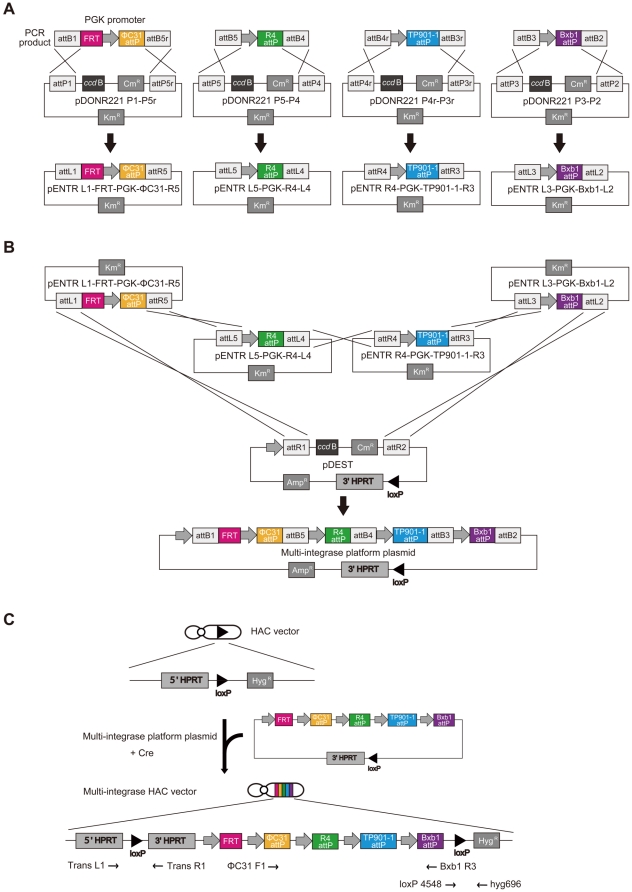
Strategy for construction of the multi-integrase HAC vector. (A) Construction of entry clones (pENTR) from gateway *attB*-flanked PCR products and donor vectors (pDONR). (B) Construction of the multi-integrase platform plasmid from four entry clones and a destination vector (pDEST). (C) Construction of the HAC vector containing the multi-integrase platform using Cre/loxP system.

The multi-integrase platform had four *attP* phage integrase recombination sites and an FRT site. The platform plasmid was constructed in *E. coli* using the Multiple Gateway system, which is useful in high-throughput construction of plasmids carrying multiple DNA sequences [44], [45]. The polymerase chain reaction (PCR) fragments for FRT-PGK-ΦC31 attP, PGK-R4 attP, PGK-TP901-1 attP and PGK-Bxb1 attP contained the appropriate gateway *attB* sites. The four fragments were recombined into four different donor vectors (pDONR™221 P1-P5r, pDONR221 P5-P4, pDONR221 P4r-P3r, pDONR221 P3-P2) to create four entry clones (pENTR L1-FRT-PGK-ΦC31-R5, pENTR L5-PGK-R4-L4, pENTR R4-PGK-TP901-1-R3, pENTR L3-PGK-Bxb1-L2) (Fig. 1A). DNA sequencing showed that 95.0% of kanamycin-resistant *E. coli* transformant colonies were correctly targeted. The four pENTR vectors and a pDEST vector were mixed in the presence of LR clonase enzyme to generate the multi-integrase platform plasmid (Fig. 1B). Using DNA sequencing, 40.7% of ampicillin-resistant *E. coli* transformant colonies were correctly targeted. The multi-integrase platform plasmid carried a loxP sequence and the 3′ hypoxanthine phosphoribosyl transferase (HPRT) sequence. The HAC vector used in this study was 21HAC1 containing the 5′ HPRT-loxP site [39]. The HPRT gene expressed in the HAC vector conferred HAT-resistance after site-specific recombination with the Cre/loxP system. The platform plasmid and Cre expression vector were co-transfected into CHO (*hprt* ^−/−^) cells carrying the HAC vector (Fig. 1C). PCR analyses using the primers (Trans L1/R1, loxP4548/hyg696 and ΦC31 F1/Bxb1 R3) showed that 50.0% HAT-resistant transfectants were correctly targeted. This ratio of circular plasmid insertion into the 5′ HPRT-loxP site on the HAC vector by HPRT gene reconstitution was similar to that observed previously (33.3%) [39]. This HAC vector was designated as the multi-integrase HAC (MI-HAC) vector. Using fluorescence *in situ* hybridization (FISH), the digoxigenin-labeled human COT1 DNA probe localized to the MI-HAC vector and the HAC vector was present as an independent minichromosome without integration into the host genome in all six randomly selected clones (data not shown). These results indicated that a multi-integrase platform can be cloned into the defined locus on the HAC vector by the Cre/loxP system.

### Site-specific recombination into the phage *attP* and FRT sites on the MI-HAC vector

**Figure 2:**
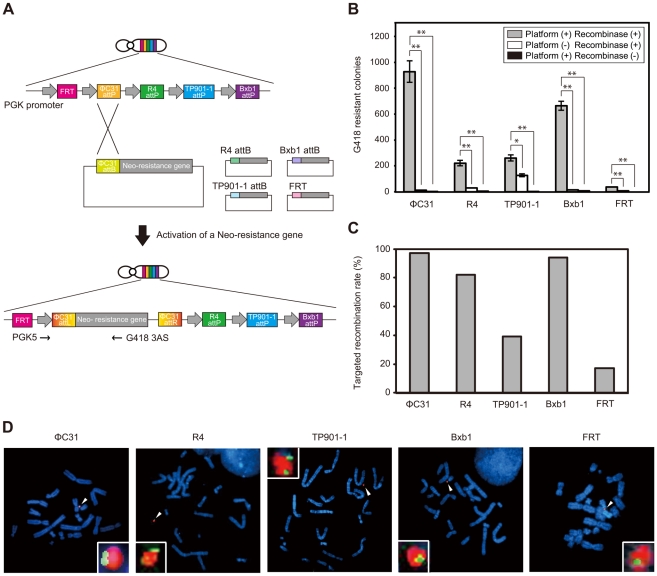
A direct comparison of the recombination efficiency mediated by phage integrases and FLPe. (A) Schematic diagram of the multi-integrase HAC system facilitating direct comparison of FLPe and phage integrase recombination activities. (B) *In vitro* integration efficiency after integrases and FLPe-mediated integration. A summary of the counted colonies for each condition after crystal violet staining is shown. Data were corrected for average colony numbers in one of the ten dishes and expressed as means ± SE. Student's *t*-test was used to determine statistically significant differences. Differences at *P*<0.05 were considered significant. *P<0.05, **P<0.01. (C) Site-specific recombination efficiency was determined by genomic PCR. (D) Fluorescence *in situ* hybridization (FISH) analysis of the recombinant multi-integrase HAC vector in CHO cells. Arrowheads indicate the dual-colored multi-integrase HAC vector during metaphase. Cells were probed with human COT-1 DNA (red) and each recombinant assay plasmid conferred fluorescence to the target cell (green).

In attempts to improve translational efficiency in mammalian cells, integrases derived from phages ΦC31, R4, TP901-1 and Bxb1 were codon-optimized. To compare the recombination efficiency of the four integrases and FLPe in an identical expression system in mammalian cells, we used the multi-integrase system based on the HAC vector. For a direct comparison of integrases and FLPe, all expression constructs were driven by the phosphoglycerate kinase 1 (PGK) promoter (Fig. 2A).

We tested whether integrases could mediate integration at phage *attP* sites more efficiently than FLPe. We also attempted to determine which integrase had the highest recombination efficiency. Recombinant assay plasmids carrying a promoterless neomycin-resistance gene with a recombination site (pNeo-ΦC31 attB, pNeo-R4 attB, pNeo-TP901-1 attB, pNeo-Bxb1 attB or pNeo-FRT) and the corresponding recombinase expression plasmids (pCMV-ΦC31, pCMV-R4, pCMV-TP901-1, pCMV-Bxb1, or pCMV-FLPe) were co-transfected into CHO cells carrying the MI-HAC vector (platform^+^/recombinase^+^, *n* = 5–9) (Fig. 2A). To ensure that the recombination event that occurred at the recombination sites on the MI-HAC vector was dependent on recombinase expression, two control experiments were performed. In one experiment, the recombinant assay plasmid was transfected without the recombinase expression plasmid into CHO cells containing the MI-HAC vector (platform^+^/recombinase^−^, *n* = 3). The second experiment involved co-transfection of the recombinase expression and recombinant assay plasmids in CHO cells containing the HAC vector without the multi-integrase platform (platform^−^/recombinase^+^, *n* = 3). In each transfection, cells were seeded into ten 10-cm tissue culture dishes at 24 h post-transfection and placed under selection with G418 at 48 h post-transfection. Cells were selected for 12 days, then assessed for colony numbers. Data were corrected for average colony numbers in one of the ten dishes (Fig. 2B). There were approximately 10–460-fold more colonies observed under the platform^+^/recombinase^+^ conditions, compared with the control conditions (platform^+^/recombinase^−^); and approximately 6–73-fold more colonies observed under the platform^+^/recombinase^+^ conditions compared with the platform^−^/recombinase^+^ transfection. These results suggested that integrases and phage *attP* sites on the MI-HAC vector were functional in CHO cells, and that these integrases yielded higher total colony numbers compared with FLPe.

To determine the frequency of the site-specific recombination event at the recombination sites on the MI-HAC vector, we employed genomic PCR analysis. Using the appropriate primers (PGK5/G418 3AS), the expected amplicon was detected in 30 of 31 (96.8%) colonies for ΦC31 integrase (Fig. 2C). In other recombinases, R4, TP901-1, Bxb1 integrases and FLPe showed average recombination frequencies of approximately 82.4, 39.3, 94.1 and 17.2%, respectively (Fig. 2C). A recombination event in the absence of the respective recombinases was not observed. FISH analyses were performed on cells to examine if each recombinant assay plasmid was inserted into the MI-HAC vector. In the pool of cells obtained from each recombinase, the FITC signal (green) indicating the recombinant assay plasmid localized at the MI-HAC vector (red; Fig. 2D). These results showed that the site-specific insertion of foreign DNA into the MI-HAC vector was achieved by integrases and FLPe.

### Generation of GFP-expressing cells with the MI-HAC vector or via random integration

**Figure 3:**
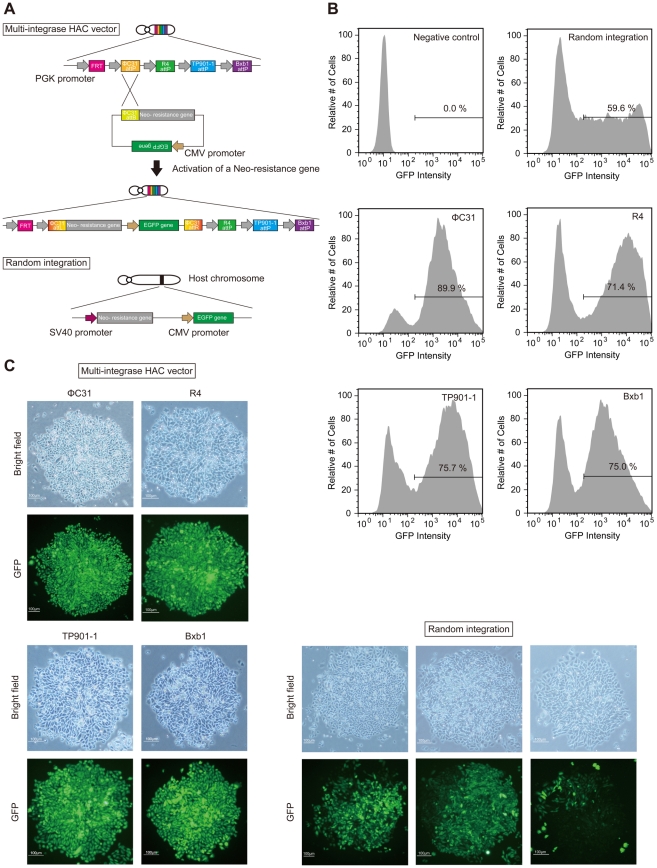
Comparison of the efficiency in generating transgenic cells. (A) Schematic diagram of the insertion of the plasmid containing the CMV promoter and the *EGFP* gene in the multi-integrase HAC vector or host chromosome. Under both conditions, cells were incubated with the reagent/DNA mixture for 24 h and selected with G418 until drug-resistant cell pools were obtained. (B) Flow cytometry analysis of the fluorescent profile of transfected CHO cells on day 12 following selection by G418 after transfection. The bars indicate the GFP-expressing cell population. (C) Expression of the inserted *EGFP* gene was tracked in cells by fluorescence microscopy. Typical images (bright field, upper panels; fluorescence, lower panels) of cells obtained by the multi-integrase HAC vector with integrases and random integration are shown. Scale bar, 100 µm.

Based on the likely activity of phage integrases in mediating recombination events in CHO cells, we tested whether the MI-HAC vector with integrases was able to produce cells expressing the gene of interest more efficiently than the conventional random integration method. The transfection experiments were carried out with pNeo-attB EGFP or pEGFP N1, which both contained the strong CMV enhancer coupled to the enhanced green fluorescent protein (*EGFP*) gene (Fig. 3A). Before transfection, the pEGFP N1 was linearized at the *Ase*I site, upstream from the CMV promoter. The host cells used in the transfection experiments were CHO cells carrying the MI-HAC vector. Each transfection was subjected to G418 selection, and colony numbers were assessed. In one such experiment, approximately 5–17-fold more colonies under G418 selective pressure for 12 days were observed when the linearized pEGFP N1 was transfected into CHO cells (random integration method), compared with pNeo-attB EGFP with the correct integrase expression plasmid (MI-HAC method). Following transfection after 12 days in G418 selection medium, the transfected cells were analyzed by flow cytometry to examine the proportion of GFP expression CHO colonies. As a result, the GFP-positive cells obtained by the MI-HAC method with ΦC31 integrase comprised 89.9% of the total cell population (Fig. 3B, Table 1). The negative control employed was CHO cells without a GFP expression cassette. Similar experiments using R4, TP901-1 and Bxb1 integrases demonstrated the proportion of GFP-positive cells to be 71.4, 75.7 and 75.0%, respectively (Fig. 3B, Table 1). Although GFP-positive cells obtained by the random integration method comprised 59.6%, there was not a great difference in the proportion of GFP-positive cells by the MI-HAC vector or the random integration methods (Table 1). However, we observed a wide distribution of GFP expression in the pool of cells obtained following application of the random integration method (Fig. 3B). In addition, we observed the expression of GFP using fluorescence microscopy in CHO colonies. As a result, in colonies obtained by the MI-HAC method, we observed homogenous GFP expression (nearly every cell expressed GFP in the clonal population) in many colonies (Fig. 3C, Table 1). In contrast, we observed only 10.0% of colonies with homogenous GFP expression after the random integration method (Table 1). Nearly 90.0% of colonies consisted of heterogenous GFP-expressing cells (a mixture of very little or no GFP-expressing cells in the clonal population) following random integration (Fig. 3C).

**Table 1:** GFP expression profiles of the transfected CHO cells.

Integrase	Proportion GFP-positive cells (%) a	Homogenous GFP expression colonies (%) b
ΦC31	89.9	87.5
R4	71.4	85.0
TP901-1	75.7	86.4
Bxb1	75.0	77.3
Random integration	59.6	10.0

a Analysis via flow cytometry.

b Analysis via fluorescence microscopy.

CHO cells obtained by the multi-integrase HAC or the random integration method were cultured in the presence of G418 and GFP expression was analyzed by flow cytometry. Additionally, the ratio of homogenous GFP-expressing colonies was evaluated by fluorescence microscopy.

### Transfer of the MI-HAC vector to mammalian cell

**Figure 4:**
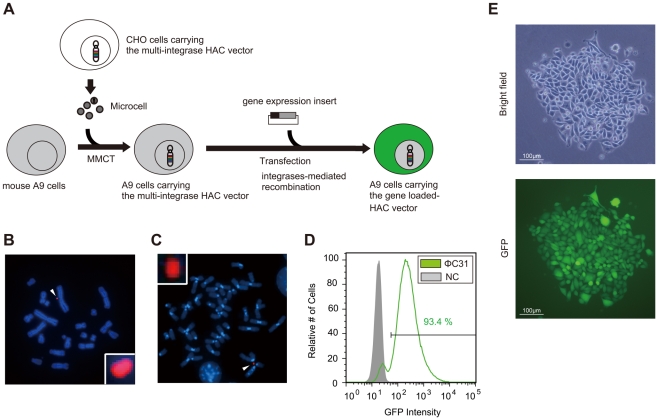
The multi-integrase HAC vector is transferable to other cell lines. (A) Microcell-mediated chromosome transfer (MMCT) of the multi-integrase HAC vector into mouse A9 cells and subsequent introduction of gene expression cassettes. (B) Typical FISH image of CHO cells carrying the multi-integrase HAC vector. The arrowhead indicates the multi-integrase HAC vector. (C) Shown is a representative metaphase spread hybridized to the human COT-1 probe (red) in A9 hybrids. (D) By flow cytometry analysis, the proportion of GFP-positive transfected A9 cells was determined. The gray curve indicates cells without a GFP expression cassette, the green curve indicates cells obtained by the multi-integrase HAC vector with ΦC31 integrase. The bar indicates the GFP-expressing cell population. (E) GFP expression was detected in the G418-resistant A9 cells by fluorescence microscopy. Representative images (bright field, upper panels; fluorescence, lower panels) of cells obtained with the multi-integrase HAC vector with ΦC31 integrase are shown. Scale bar, 100 µm.

An important application of the MI-HAC vector is its potential use as a vector for generation of transgenic cells. At present, the most commonly used method to introduce HACs into recipient target cells is microcell-mediated chromosome transfer (MMCT). As assessed by FISH using the human-specific COT1 probe, CHO cells possessed an independently segregating mini-chromosome corresponding to the MI-HAC vector (Fig. 4B). The MI-HAC vector was introduced into mouse A9 cells by MMCT. After selection with hygromycin B, eight clones were obtained. We confirmed that these drug-resistant A9 clones contained the MI-HAC vector by genomic PCR, with FISH analysis revealing that the MI-HAC vector was segregated independently, with neither host genome insertion nor translocation in all eight clones (Fig. 4C). Next, we examined whether an integrase was functional in another mammalian cell line, mouse A9. Similar experiments in CHO cells using ΦC31 integrase and the GFP expression plasmid were carried out in the A9 cells. After 12 days in G418 selection medium, flow cytometry analysis of these cell populations was conducted. Consequently, the GFP-positive cells acquired by the MI-HAC method with the ΦC31 integrase comprised 93.4% of the total cell population (Fig. 4D). The negative control employed was A9 cells without a GFP expression cassette. Similar to the CHO cells, we observed homogenous GFP expression in most A9 colonies (Fig. 4E).

## Discussion

In this study, we constructed a HAC vector with multi-integrase recombination sites. We also demonstrate that phage integrases, ΦC31, R4, TP901-1 and Bxb1, mediated recombination efficiently between att sites; and that *de novo* integrases combined with the HAC vector to produce transgenic cells more efficiently than conventional methods in mammalian cells. It was also shown that the MI-HAC vector was transferable to cultured cell lines by MMCT.

The recombination activity for ΦC31, R4, TP901-1 and Bxb1 integrases was assessed in CHO cells carrying the MI-HAC vector. The results of these recombination assays indicated that these integrases conferred higher recombination activity (39.3–96.8%) in mammalian cells compared with FLPe (17.2%). However, whether codon optimization of integrases gave high recombination activity was not tested. These integrases may be better suited for cassette exchange reactions as compared with Cre or FLP [18]. The proportion of plasmid insertion into the phage *attP* sites on the MI-HAC vector by neomycin gene reconstitution (39.3–96.8%) was higher compared with insertion into the 5′ HPRT-loxP site of the 21HAC1 by HPRT reconstitution (33.3%) or into the 3′ neo-loxP site of the 21ΔqHAC/21ΔpqHAC by neomycin gene reconstitution (37.5%) [38], [39], [46].

To increase the speed and efficiency of generating transgenic cells, the MI-HAC system combined with integrases was applied. This MI-HAC system consists of four main components: the MI-HAC vector, the MI-HAC cell line, the gene insertion plasmid and the integrase expression plasmid. We revealed that the MI-HAC method combined with integrases allows us to obtain transgenic cells more efficiently than the random integration method. Based on these favorable results from the MI-HAC system, in particular with ΦC31 integrase, it was proposed that the cell selection process could be omitted altogether. The phenomenon of heterocellular transgene expression in a clonal subpopulation of cells occurred rarely *via* the MI-HAC method as compared with the random integration method. As reported previously, the HAC vector enables stable expression of the inserted gene, and is not affected by position in the genome [38], [39], [47]. Our data suggested a genomic targeting system using the MI-HAC vector with integrases enabled transgenes to be targeted to a pre-determined locus, thereby limiting the effects of position and allowing transgenic cells to be generated more efficiently and robustly than the random integration method. These results are specific for the MI-HAC vector, the likelihood of these results carrying over to normal mammalian chromosomes was not tested.

Generation of cell lines often requires the use of cell clones containing stably integrated transgenes. Conventional methods such as random integration can lead to a broad variation in expression profiles between cells due to the differences in variable position effects on the function of a specific transgene. Another approach is generation of site-specific targeting cell lines using ΦC31 and R4 integrases [48]. However, this approach requires confirmation that the R4 plasmid inserted locus is not subject to genomic positional effects. Recent studies have found that aberrant events, including chromosomal rearrangements in the target cell genome, were induced by the ΦC31 and R4 integrases [49], [50], [51], [52]. In contrast, due to the transfer ability of the MI-HAC vector, effects such as chromosomal rearrangement do not occur with the phage integrases. We demonstrated the transfer of the MI-HAC vector from CHO cells to mouse A9 cells by MMCT. We previously demonstrated that the HAC vector can be transferred into various cell types, such as mouse embryonic stem cells, human primary fibroblasts, as well as hematopoietic and mesenchymal stem cells [41], [47]. We confirmed thatΦC31integrase was functional in mouse A9 cells, and the MI-HAC vector with ΦC31integrase can be applied as an efficient gene expression system in various cell environments.

In addition to chromosomal integration events, there are certain instances where chromosomal deletion or excision is desired, such as in the generation of knockout or conditional knockout transgenic animals. One of the techniques to accomplish deletion or excision of a sequence of interest is through the use of site-specific recombinases [53]. Recent studies have reported that phage integrases, such as ΦC31, R4 and TP901-1, were functional in the mammalian embryonic environment and had the potential for creation of transgenic animals and modification of the mammalian genome *in vivo* [26], [54], [55].

In this study, we combined phage integrase-mediated recombination technology with the HAC vector. The MI-HAC system is able to efficiently and precisely carry out recombination in mammalian cells, thereby making it a valuable unique genetic tool for investigating gene function, gene therapy and animal transgenesis.

## Materials and Methods

### Construction of the multi-integrase platform plasmid

The multi-integrase platform plasmid was constructed using the Multisite Gateway™ kit (Invitrogen, Carlsbad, CA, USA) as previously described [44], [45]. The PCR fragments for FRT-PGK-ΦC31 attP, PGK-R4 attP, PGK-TP901-1 attP and PGK-Bxb1 attP (Table S1) flanked by the appropriate gateway *attB* sites were created in two steps. First, PGK-hyg (Clontech, Palo Alto, CA, USA) was used as a template for PCR amplification with primer pair F1 and R1 (Table S2). These products then served as templates for the second PCR amplification step with the primer pair F1 and R2 (F2 and R2 for FRT-PGK-ΦC31 attP; Table S2). The gateway *attB*-flanked PCR product was recombined with a donor vector containing the corresponding gateway *attP* signals in a BP reaction to generate an entry clone. Approximately 150 ng of PCR fragment and a donor vector (pDONR221 P1-P5r, pDONR 221 P5-P4, pDONR 221 P4r-P3r, pDONR 221 P3-P2) were mixed with 2 µl of BP clonase™ II enzyme mix, and adjusted to 10 µl with TE buffer. The mixture was then incubated at 25°C for 1 h to create four entry clones (pENTR L1-FRT-PGK-ΦC31-R5, pENTR L5-PGK-R4-L4, pENTR R4-PGK-TP901-1-R3, pENTR L3-PGK-Bxb1-L2). After the BP reaction, the enzymes were inactivated by treatment with Proteinase K for 10 min at 37°C. Competent Mach1™ T1^R^ *E. coli* (Invitrogen) was used according to the supplier's instruction. After transformation, the cell solution was diluted with SOC medium and incubated at 37°C for 1 h. The transformation reactions were then spread onto LB agar plates containing 50 µg/ml kanamycin (Km) and incubated for 14 h at 37°C. The sequence of the four pENTRs was confirmed by DNA sequencing. The destination vector (pDEST) contained the R1-*ccd*B-Cm^R^-R2 cassette (Invitrogen), a single PGK promoter and loxP-3′ HPRT cassette. The backbone vector was isolated by blunt PCR from the plasmid V901-3′HPRT-loxP [39] using the primers PGK2362 and loxP4548. The R1-*ccd*B-Cm^R^-R2 cassette was ligated into the blunt V901-3′HPRT-loxP plasmid. To construct the destination vector containing a toxic *ccd*B gene, *ccd*B survival™ competent cells (Invitrogen) were used for propagation according to the supplier's instruction. After transformation, the cell solution was diluted with SOC medium and incubated at 37°C for 1 h. The transformation reactions were then spread onto LB agar plates containing 20 µg/ml chloramphenicol (Cm) and incubated for 18 h at 37°C. Sequence and orientation of pDEST and the R1-*ccd*B-Cm^R^-R2 cassette was confirmed by DNA sequencing. The four entry clones were recombined with a destination vector to generate the multi-integrase platform plasmid. Before this reaction, the pDEST was linearized at the *Bgl*II site, between a *ccd*B gene and a Cm^R^ gene. Around 20 ng of each entry clone and 80 ng of destination vector were mixed with 2 µl of LR clonase II Plus enzyme mix, and made up to 10 µl with TE buffer. The mixture was then incubated at 25°C for 16 h. After this reaction, the enzyme was inactivated by treatment with Proteinase K for 10 min at 37°C. Competent Mach1 T1^R^ *E. coli* were used for transformation. Following transformation, the cell solution was diluted with SOC medium and incubated at 37°C for 1 h. The transformation reactions were then spread onto LB agar plates containing 100 µg/ml ampicillin (Amp) and incubated for 14 h at 37°C. The multi-integrase plasmid carried the non-functional loxP-3′ HPRT for targeting the HAC vector. Cre recombinase-catalyzed integration required a functional HPRT gene.

### DNA constructs

The mammalian codon-optimized integrase expression plasmids, pCMV-ΦC31, pCMV-R4, pCMV-TP901-1 and pCMV-Bxb1 were created. The coding sequences of ΦC31, R4, TP901-1 and Bxb1 integrase were synthesized *de novo* (ΦC31: Codon device, Cambridge, MA, USA; others, Invitrogen) based on the published ΦC31, R4, TP901-1 and Bxb1 integrase coding sequences (GenBank accession numbers: ΦC31, CAA07153; R4, BAA07372; TP901-1, CAA59475; Bxb1, AAG59740). The native amino acid sequence but with mammalian codon usage of *de novo* synthesized integrases are shown in Figures S1, S2, S3 and S4. The ΦC31, R4, TP901-1 and Bxb1 coding sequences were cloned into mammalian expression vectors pVAX1 (Invitrogen) driven by the high expressing CMV promoter. The synthesized sequence of ΦC31 integrase was digested with *Kpn*I and *Xba*I and cloned into the *Kpn*I and *Xba*I sites of pVAX1. The R4, TP901-1 and Bxb1 integrases were digested with *Nhe*I and *Xho*I and cloned into the equivalent sites of pVAX1. The FLPe expression vector pOG44 (Invitrogen) was used as the backbone for pCMV-FLPe.

Recombinant assay plasmids were used for integrase- or FLPe-mediated recombination assays in CHO cells. This plasmid carried the ΦC31, R4, TP901-1, Bxb1 *attB* or FRT sites (Table S1) positioned at the 5′ end of a neomycin-resistance gene (pNeo-ΦC31 attB, pNeo-R4 attB, pNeo-TP901-1 attB, pNeo-Bxb1 attB or pNeo-FRT). The neomycin-resistance gene was isolated by blunt PCR from pIRES Neo2 (Clontech, Mountain View, CA, USA) using the primers Neo F and Neo R. This PCR product was blunt cloned into the *Eco*RV/*Sma*I-digested pSLR-test (Toyobo, Osaka, Japan), replacing the *SLR* gene and creating the plasmid pNeo. Sequence and orientation of a neomycin-resistance gene was confirmed by DNA sequencing. The ΦC31 *attB*, Bxb1 *attB* and FRT site was synthesized by Integrated DNA technologies Inc. (Coralville, IA, USA). The R4 *attB* and TP901-1 *attB* sites were synthesized by Invitrogen. The ΦC31 *attB* and R4 *attB* sites were digested with *Sal*I and cloned into the *Sal*I site of pNeo. The TP901 *attB* and FRT site was digested using *Cla*I and cloned into the equivalent site of pNeo. The Bxb1 *attB* site was digested with *Nhe*I and cloned into the *Nhe*I-digested pNeo. Orientation of each of the *attB* and FRT sites was confirmed by DNA sequencing.

The plasmid pNeo-attB EGFP was used for GFP expression cell assays in mammalian cells. The CMV promoter and *EGFP* gene were amplified by PCR from the pEGFP N1 (Clontech) lacking the *Bam*HI recognition sequence in the multi-cloning site using the primers CMV-GFP F and CMV-GFP R. The PCR product was digested with *Bam*HI and cloned into the equivalent site of pNeo-attB, creating pNeo-ΦC31 attB EGFP, pNeo-R4 attB EGFP, pNeo-TP901-1 attB EGFP and pNeo-Bxb1 attB EGFP. Sequence and orientation of the CMV promoter and *EGFP* gene were confirmed by DNA sequencing.

### Cell culture and DNA transfection

CHO cells carrying the HAC vector were maintained at 37°C in Ham's F-12 nutrient mixture (Invitrogen) supplemented with 10% fetal bovine serum (FBS; JRH Biosciences, Lenexa, KS, USA). The HAC vector used in this study was 21HAC1 [39]. Mouse A9 cells used as a fusion recipient for chromosome transfer were maintained at 37°C in Dulbecco's modified Eagle's medium (DMEM; Sigma, St Louis, MO, USA) plus 10% FBS. A9 hybrids carrying the MI-HAC vector were selected in a growth medium containing 800 µg/ml hygromycin B (Wako, Osaka, Japan).

For DNA transfection to construct the MI-HAC vector, 1×10^6^ HPRT-deficient CHO cells containing the HAC vector were seeded in wells of 6-well tissue culture plates 24 h before transfection. Approximately 3 µg multi-integrase platform plasmid carrying loxP and 3′HPRT were co-transfected with 1 µg Cre-expression plasmid pBS185 (Invitrogen) using Lipofectamine2000 (Invitrogen). CHO clones containing the HAC vector in which site-specific insertion of the multi-integrase plasmid had occurred were selected using HAT (Sigma) over a period of 12 days.

### Genomic PCR analyses

Genomic DNA from cell lines was extracted using a genomic extraction kit (Sigma). PCR analyses were carried out using standard techniques. The primer pairs used for confirmation of the multi-integrase platform were ΦC31 F1 and Bxb1 R3. The primer pairs used for detection of HPRT gene reconstitution were Trans L1 and Trans R1, along with loxP4548 and hyg696. The primer pairs utilized for detection of neomycin gene reconstitution were PGK5 and G418 3AS.

### Fluorescence in situ hybridization (FISH)

FISH analysis of CHO and A9 cells was performed with either fixed metaphase or interphase nuclei using digoxigenin-labeled (Roche, Basel, Switzerland) human COT1 DNA (Invitrogen) and biotin-labeled DNA (pNeo-ΦC31 attB, pNeo-R4 attB, pNeo-TP901-1 attB, pNeo-Bxb1 attB or pNeo-FRT) as described previously [56]. Chromosomal DNA was counterstained with DAPI (Sigma). The images were captured using the Argus system (Hamamatsu Photonics, Hamamatsu, Japan) or NIS elements (Nikon, Tokyo, Japan).

### Assay for integrase-mediated recombination on the MI-HAC vector

For DNA transfection, 1×10^6^ CHO cells containing the MI-HAC vector were seeded into wells of 6-well tissue culture plates 24 h before transfection. CHO cells carrying the MI-HAC vector were co-transfected with 3 µg recombinant assay plasmid and 1 µg the corresponding recombinase expression plasmid (platform^+^/recombinase^+^) using Lipofectamine2000 (Invitrogen). As a negative control for integration into the MI-HAC vector, parallel transfections were carried out with a recombinant assay plasmid lacking a recombinase expression plasmid (platform^+^/recombinase^−^). CHO cells containing the HAC vector without the multi-integrase platform were co-transfected with a recombinant assay plasmid and a recombinase expression plasmid (platform^−^/recombinase^+^). The cells were seeded into ten 10-cm dishes at 24 h post-transfection and placed under selection with 600 µg/ml G418 (Invitrogen) at 48 h post-transfection. Cells were selected for 12 days, then assessed for colony numbers through the use of crystal violet dye. Solutions containing 1% crystal violet (Merck KGaA, Darmstadt, Germany) with 70% ethanol (Wako, Osaka, Japan) were used to simultaneously fix and stain surviving cells to make them easily visible.

For the GFP expression assay, CHO or A9 cells containing the MI-HAC vector were co-transfected using pNeo-attB-EGFP with the corresponding integrase expression plasmid. At 24 h post-transfection, the cells were seeded into three 10-cm dishes, and at 48 h post-transfection the medium was replaced with F12 containing 600 µg/ml G418 or DMEM supplemented with 800 µg/ml G418. Selective pressure was maintained for 12 days, after which the G418-resistant colonies were analyzed by flow cytometry and observed using fluorescence microscopy. To compare with the MI-HAC method, parallel transfections were done with the linearized pEGFP N1. Before transfection, the pEGFP N1 was linearized at the *Ase*I site upstream of the CMV promoter.

### Flow cytometry

The ratio of cells expressing GFP was determined, and transfected cells under selective pressure were analyzed with a Moflo XDP (Coulter, Fullerton, CA, USA). To exclude non-viable cells in our experiments, all samples were stained with propidium iodide (PI). Between 23,000–26,000 events were acquired for each sample. The GFP and PI were excited with a 488 nm argon laser, and were detected with band-pass filters of 530/40 nm and 613/20 nm, respectively. To set the parameters for flow cytometry analysis, non-transfected cells were used as negative controls. Flow cytometry data analysis and figure generation was performed using FlowJo software (Tree Star Inc, Ashland, OR, USA).

### Microcell-mediated chromosome transfer (MMCT)

MMCT was performed according to standard protocols [56], [57]. The MI-HAC vector was transferred from CHO cells into A9 cells using MMCT. Briefly, microcells were prepared by centrifuging the CHO cells and fusing 5×10^6^ A9 cells with 47% polyethylene glycol 1000 (Wako). The A9 hybrids were selected with 800 µg/ml hygromycin B (Wako) and picked for expansion.

## Supporting Information

Figure S1 **Nucleotide sequence of mammalian codon-optimized ΦC31 integrase.** The nucleotide sequence of ΦC31 integrase used in this study. A mammalian codon-optimized ΦC31 integrase gene was synthesized *de novo* according to the native ΦC31 integrase amino acid sequence.
(DOC)

Figure S2 **Nucleotide sequence of the mammalian codon-optimized R4 integrase.** The nucleotide sequence of the R4 integrase used in this study. A mammalian codon-optimized R4 integrase gene was synthesized *de novo* according to the native R4 integrase amino acid sequence.
(DOC)

Figure S3 **Nucleotide sequence of mammalian codon-optimized TP901-1 integrase.** The nucleotide sequence of TP901-1 integrase used in this study. A mammalian codon-optimized TP901-1 integrase gene was synthesized *de novo* according to the native TP901-1 integrase amino acid sequence.
(DOC)

Figure S4 **Nucleotide sequence of mammalian codon-optimized Bxb1 integrase.** The nucleotide sequence of the Bxb1 integrase used in this study. A mammalian codon-optimized Bxb1 integrase gene was synthesized *de novo* according to the native Bxb1 integrase amino acid sequence.
(DOC)

Table S1 Sequence of the attP, attB and FRT sites used in this study.
(XLS)

Table S2 The PCR primers used in this study.
(XLS)
