# Supplementary material for: A Method for Producing Transgenic Cells Using a Multi-Integrase System on a Human Artificial Chromosome Vector
Source: PLoS One. 2011 Feb 24;6(2):e17267. doi: 10.1371/journal.pone.0017267 (PMC3044732; doi:10.1371/journal.pone.0017267)
Supplement: Figure S1 — Nucleotide sequence of mammalian codon-optimized ΦC31 integrase. The nucleotide sequence of ΦC31 integrase used in this study. A mammalian codon-optimized ΦC31 integrase gene was synthesized de novo according to the native ΦC31 integrase amino acid sequence. (DOC) [file pone.0017267.s001.doc]

1 ATG GAC ACC TAC GCC GGC GCT TAC GAT AGA CAG AGC AGG GAG CGG GAA AAC AGC TCT GCT 61

M D T Y A G A Y D R Q S R E R E N S S A

61 GCA AGT CCT GCT ACA CAA CGG AGC GCC AAC GAG GAC AAG GCC GCA GAT CTG CAG AGG GAG 121

A S P A T Q R S A N E D K A A D L Q R E

121 GTG GAG CGT GAC GGG GGA AGG TTC CGG TTT GTC GGT CAC TTC AGC GAA GCA CCG GGG ACT 181

V E R D G G R F R F V G H F S E A P G T

181 AGT GCC TTT GGG ACC GCT GAG CGC CCA GAA TTT GAG AGG ATA CTG AAC GAA TGC AGA GCC 241

S A F G T A E R P E F E R I L N E C R A

241 GGA AGG CTG AAT ATG ATT ATC GTG TAC GAC GTC AGT CGG TTT TCA CGC CTG AAG GTT ATG 301

G R L N M I I V Y D V S R F S R L K V M

301 GAT GCA ATT CCC ATC GTG TCT GAA CTG CTC GCA CTG GGT GTC ACA ATT GTG AGC ACT CAG 361

D A I P I V S E L L A L G V T I V S T Q

361 GAA GGC GTT TTC AGG CAA GGG AAT GTT ATG GAC CTG ATT CAT CTG ATT ATG CGC CTC GAT 421

E G V F R Q G N V M D L I H L I M R L D

421 GCA TCC CAT AAG GAA TCT TCA CTC AAG AGT GCA AAA ATC CTG GAT ACC AAG AAT CTC CAG 481

A S H K E S S L K S A K I L D T K N L Q

481 CGT GAA CTG GGG GGC TAC GTG GGA GGC AAA GCC CCG TAC GGA TTT GAA CTG GTC AGT GAG 541

R E L G G Y V G G K A P Y G F E L V S E

541 ACT AAA GAA ATT ACT CGA AAT GGC CGA ATG GTG AAC GTT GTG ATA AAC AAA CTG GCT CAC 601

T K E I T R N G R M V N V V I N K L A H

601 AGC ACT ACC CCT CTC ACA GGT CCC TTC GAG TTT GAA CCT GAC GTG ATT AGA TGG TGG TGG 661

S T T P L T G P F E F E P D V I R W W W

661 AGG GAG ATC AAG ACA CAT AAA CAT CTG CCC TTT AAA CCC GGC AGC CAG GCC GCT ATC CAC 721

R E I K T H K H L P F K P G S Q A A I H

721 CCA GGG AGT ATT ACA GGC CTG TGT AAA CGA ATG GAC GCA GAC GCC GTC CCA ACC CGA GGT 781

P G S I T G L C K R M D A D A V P T R G

781 GAA ACT ATT GGG AAG AAA ACC GCA AGT AGC GCT TGG GAC CCC GCA ACC GTG ATG CGA ATA 841

E T I G K K T A S S A W D P A T V M R I

841 CTC CGA GAT CCT AGG ATC GCA GGA TTT GCC GCT GAG GTT ATT TAC AAG AAG AAG CCA GAT 901

L R D P R I A G F A A E V I Y K K K P D

901 GGC ACA CCT ACT ACC AAA ATC GAA GGT TAC CGG ATT CAA CGC GAC CCT ATC ACT CTG AGG 961

G T P T T K I E G Y R I Q R D P I T L R

961 CCT GTT GAA CTC GAC TGC GGG CCC ATT ATC GAA CCC GCC GAA TGG TAC GAG CTC CAG GCA 1021

P V E L D C G P I I E P A E W Y E L Q A

1021 TGG CTC GAT GGC CGT GGT AGG GGC AAG GGG CTG TCT CGC GGG CAG GCT ATT CTG AGT GCT 1081

W L D G R G R G K G L S R G Q A I L S A

1081 ATG GAT AAA CTG TAC TGT GAA TGC GGC GCA GTT ATG ACT TCC AAA AGG GGA GAG GAA AGT 1141

M D K L Y C E C G A V M T S K R G E E S

1141 ATT AAA GAT TCT TAT AGA TGT CGA CGG CGA AAG GTG GTC GAC CCC TCC GCT CCC GGG CAA 1201

I K D S Y R C R R R K V V D P S A P G Q

1201 CAC GAG GGA ACT TGC AAC GTG TCC ATG GCT GCC CTC GAT AAG TTC GTG GCT GAA CGG ATC 1261

H E G T C N V S M A A L D K F V A E R I

1261 TTT AAC AAG ATC AGA CAC GCC GAG GGC GAT GAA GAG ACT CTG GCT CTC CTG TGG GAG GCT 1321

F N K I R H A E G D E E T L A L L W E A

1321 GCC AGG CGG TTC GGT AAA CTG ACA GAG GCC CCC GAG AAG TCT GGG GAG CGC GCA AAT CTG 1381

A R R F G K L T E A P E K S G E R A N L

1381 GTT GCC GAA AGG GCT GAC GCA CTG AAC GCT CTG GAA GAG CTG TAC GAG GAT AGG GCC GCA 1441

V A E R A D A L N A L E E L Y E D R A A

1441 GGA GCT TAC GAC GGC CCA GTC GGT CGA AAA CAC TTT CGG AAG CAG CAG GCT GCA CTC ACT 1501

G A Y D G P V G R K H F R K Q Q A A L T

1501 CTG CGC CAA CAG GGT GCA GAG GAA AGA CTG GCC GAG CTG GAA GCC GCA GAG GCT CCC AAA 1561

L R Q Q G A E E R L A E L E A A E A P K

1561 CTG CCA CTC GAC CAG TGG TTT CCC GAG GAT GCT GAT GCA GAC CCC ACA GGC CCT AAG TCC 1621

L P L D Q W F P E D A D A D P T G P K S

1621 TGG TGG GGC AGG GCT TCA GTT GAC GAT AAA CGC GTT TTC GTT GGG CTG TTT GTG GAC AAA 1681

W W G R A S V D D K R V F V G L F V D K

1681 ATT GTG GTT ACT AAG AGT ACC ACT GGC AGA GGA CAA GGC ACC CCA ATC GAG AAA CGC GCC 1741

I V V T K S T T G R G Q G T P I E K R A

1741 TCT ATT ACA TGG GCA AAA CCG CCA ACC GAC GAT GAC GAA GAT GAC GCT CAG GAC GGT ACT 1801

S I T W A K P P T D D D E D D A Q D G T

1801 GAG GAT GTC GCC GCA TCT AGA GGG CCC GTT TAA 1833

E D V A A S R G P V *
