# Supplementary material for: A Method for Producing Transgenic Cells Using a Multi-Integrase System on a Human Artificial Chromosome Vector
Source: PLoS One. 2011 Feb 24;6(2):e17267. doi: 10.1371/journal.pone.0017267 (PMC3044732; doi:10.1371/journal.pone.0017267)
Supplement: Figure S2 — Nucleotide sequence of the mammalian codon-optimized R4 integrase. The nucleotide sequence of the R4 integrase used in this study. A mammalian codon-optimized R4 integrase gene was synthesized de novo according to the native R4 integrase amino acid sequence. (DOC) [file pone.0017267.s002.doc]

1 ATG AAC AGA GGT GGT CCT ACA GTG AGA GCT GAT ATT TAC GTA CGT ATC AGC CTG GAT AGA 61

M N R G G P T V R A D I Y V R I S L D R

61 ACC GGT GAA GAA CTC GGT GTA GAA AGA CAA GAA GAA AGC TGC AGA GAA CTC TGC AAG TCC 121

T G E E L G V E R Q E E S C R E L C K S

121 CTG GGT ATG GAA GTT GGT CAA GTC TGG GTA GAT AAC GAT CTG TCA GCA ACA AAA AAG AAT 181

L G M E V G Q V W V D N D L S A T K K N

181 GTG GTG AGA CCA GAT TTC GAA GCC ATG ATT GCC AGC AAT CCT CAA GCA ATT GTC TGT TGG 241

V V R P D F E A M I A S N P Q A I V C W

241 CAC ACA GAT CGT CTC ATA CGA GTG ACT CGT GAT CTG GAA CGC GTG ATC GAT CTG GGT GTG 301

H T D R L I R V T R D L E R V I D L G V

301 AAC GTC CAT GCA GTT ATG GCC GGT CAT CTG GAT CTC TCA ACT CCC GCC GGT CGT GCT GTG 361

N V H A V M A G H L D L S T P A G R A V

361 GCT AGA ACT GTT ACG GCA TGG GCA ACC TAT GAA GGT GAA CAA AAG GCT GAA CGT CAA AAA 421

A R T V T A W A T Y E G E Q K A E R Q K

421 CTC GCC AAC ATC CAA AAC GCC CGC GCT GGT AAA CCC TAC ACT CCA GGT ATT CGC CCT TTC 481

L A N I Q N A R A G K P Y T P G I R P F

481 GGT TAT GGT GAT GAT CAT ATG ACA ATC GTA ACC GCT GAA GCT GAT GCC ATT AGA GAT GGT 541

G Y G D D H M T I V T A E A D A I R D G

541 GCC AAG ATG ATC CTC GAT GGT TGG AGT CTG TCT GCT GTT GCC CGC TAT TGG GAA GAA CTC 601

A K M I L D G W S L S A V A R Y W E E L

601 AAG CTG CAA AGC CCC AGA AGC ATG GCC GCC GGT GGT AAG GGT TGG TCA CTC CGC GGT GTC 661

K L Q S P R S M A A G G K G W S L R G V

661 AAA AAA GTG CTG ACC TCA CCA CGT TAC GTG GGT CGA AGC AGC TAT TTG GGT GAA GTG GTC 721

K K V L T S P R Y V G R S S Y L G E V V

721 GGT GAT GCA CAA TGG CCA CCC ATC CTC GAT CCC GAT GTG TAT TAC GGT GTT GTC GCC ATC 781

G D A Q W P P I L D P D V Y Y G V V A I

781 CTC AAC AAC CCC GAT CGA TTC TCA GGT GGT CCA CGA ACT GGT AGA ACA CCC GGT ACT CTG 841

L N N P D R F S G G P R T G R T P G T L

841 TTG GCT GGT ATC GCC CTG TGT GGT GAA TGC GGT AAG ACT GTG TCA GGT CGA GGT TAT CGT 901

L A G I A L C G E C G K T V S G R G Y R

901 GGT GTC CTG GTG TAT GGT TGC AAA GAT ACT CAC ACC CGC ACT CCC CGA TCA ATT GCA GAT 961

G V L V Y G C K D T H T R T P R S I A D

961 GGT CGC GCA AGC AGC AGT ACT TTG GCA CGT CTT ATG TTC CCC GAT TTT CTC CCT GGT CTG 1021

G R A S S S T L A R L M F P D F L P G L

1021 CTC GCT AGT GGT CAA GCC GAA GAT GGT CAA TCA GCA GCC TCC AAG CAC TCA GAA GCT CAA 1081

L A S G Q A E D G Q S A A S K H S E A Q

1081 ACA CTT AGA GAA AGA CTT GAT GGT CTG GCC ACA GCT TAC GCA GAA GGT GCT ATC TCT CTG 1141

T L R E R L D G L A T A Y A E G A I S L

1141 AGT CAA ATG ACC GCA GGT TCA GAA GCC CTC CGC AAG AAG CTG GAA GTT ATC GAA GCT GAT 1201

S Q M T A G S E A L R K K L E V I E A D

1201 CTC GTC GGT TCA GCC GGT ATT CCA CCT TTC GAT CCA GTC GCT GGT GTG GCC GGT CTC ATC 1261

L V G S A G I P P F D P V A G V A G L I

1261 AGC GGT TGG CCC ACC ACT CCT CTG CCA ACT CGC AGA GCT TGG GTT GAT TTC TGC CTT GTT 1321

S G W P T T P L P T R R A W V D F C L V

1321 GTG ACA CTG AAC ACA CAA AAG GGT AGA CAC GCC TCC TCC ATG ACA GTG GAT GAT CAC GTT 1381

V T L N T Q K G R H A S S M T V D D H V

1381 ACT ATT GAA TGG CGA GAT GTC GCC GAA TGA 1410

T I E W R D V A E *
