# Supplementary material for: A Method for Producing Transgenic Cells Using a Multi-Integrase System on a Human Artificial Chromosome Vector
Source: PLoS One. 2011 Feb 24;6(2):e17267. doi: 10.1371/journal.pone.0017267 (PMC3044732; doi:10.1371/journal.pone.0017267)
Supplement: Figure S3 — Nucleotide sequence of mammalian codon-optimized TP901-1 integrase. The nucleotide sequence of TP901-1 integrase used in this study. A mammalian codon-optimized TP901-1 integrase gene was synthesized de novo according to the native TP901-1 integrase amino acid sequence. (DOC) [file pone.0017267.s003.doc]

1 ATG ACA AAG AAA GTG GCA ATC TAT ACC AGA GTG AGC ACC ACA AAC CAA GCA GAA GAA GGT 61

M T K K V A I Y T R V S T T N Q A E E G

61 TTC TCT ATT GAT GAA CAA ATA GAT CGT TTG ACC AAA TAT GCC GAA GCT ATG GGT TGG CAA 121

F S I D E Q I D R L T K Y A E A M G W Q

121 GTG TCT GAT ACA TAT ACA GAT GCC GGT TTC TCT GGT GCT AAG CTG GAA CGC CCT GCT ATG 181

V S D T Y T D A G F S G A K L E R P A M

181 CAA AGA CTG ATT AAT GAT ATC GAA AAC AAA GCA TTT GAT ACA GTG CTG GTA TAC AAA CTG 241

Q R L I N D I E N K A F D T V L V Y K L

241 GAT AGA CTG AGC AGA TCC GTC AGA GAT ACA CTG TAT CTG GTG AAG GAT GTA TTC ACA AAG 301

D R L S R S V R D T L Y L V K D V F T K

301 AAT AAG ATT GAT TTT ATT TCT CTC AAT GAA TCC ATC GAT ACA AGT TCC GCT ATG GGT TCA 361

N K I D F I S L N E S I D T S S A M G S

361 CTG TTC CTC ACA ATC TTG TCC GCC ATA AAC GAA TTT GAA CGT GAA AAC ATT AAG GAA AGA 421

L F L T I L S A I N E F E R E N I K E R

421 ATG ACT ATG GGT AAG CTG GGT CGC GCA AAA TCT GGT AAA AGC ATG ATG TGG ACT AAA ACA 481

M T M G K L G R A K S G K S M M W T K T

481 GCA TTT GGT TAC TAC CAC AAT AGA AAG ACA GGT ATC CTG GAA ATT GTG CCA CTG CAA GCT 541

A F G Y Y H N R K T G I L E I V P L Q A

541 ACT ATC GTT GAA CAA ATC TTC ACC GAT TAC TTG TCT GGT ATT TCC CTG ACT AAG CTC CGT 601

T I V E Q I F T D Y L S G I S L T K L R

601 GAT AAA CTC AAC GAA TCT GGT CAC ATC GGT AAG GAT ATC CCC TGG TCC TAT AGA ACT CTG 661

D K L N E S G H I G K D I P W S Y R T L

661 AGA CAA ACC CTG GAT AAT CCT GTA TAC TGC GGT TAC ATC AAG TTC AAA GAT TCA TTG TTC 721

R Q T L D N P V Y C G Y I K F K D S L F

721 GAA GGT ATG CAC AAG CCA ATC ATC CCC TAC GAA ACA TAC CTC AAG GTA CAA AAG GAA CTG 781

E G M H K P I I P Y E T Y L K V Q K E L

781 GAA GAA CGA CAA CAA CAA ACA TAC GAA CGT AAC AAC AAT CCC CGC CCA TTT CAA GCC AAA 841

E E R Q Q Q T Y E R N N N P R P F Q A K

841 TAC ATG TTG TCC GGT ATG GCT AGA TGC GGT TAC TGC GGT GCA CCC TTG AAA ATC GTT CTC 901

Y M L S G M A R C G Y C G A P L K I V L

901 GGT CAC AAG CGT AAG GAT GGT TCT CGA ACT ATG AAG TAC CAC TGC GCA AAT AGA TTC CCA 961

G H K R K D G S R T M K Y H C A N R F P

961 CGA AAA ACG AAG GGT ATC ACT GTA TAC AAC GAT AAC AAA AAG TGC GAT TCC GGT ACA TAT 1021

R K T K G I T V Y N D N K K C D S G T Y

1021 GAT CTC AGC AAT CTT GAA AAT ACG GTT ATT GAT AAC CTT ATT GGT TTC CAA GAA AAT AAT 1081

D L S N L E N T V I D N L I G F Q E N N

1081 GAT TCC CTG CTG AAG ATC ATC AAT GGT AAC AAT CAA CCC ATC TTG GAT ACC AGT TCA TTC 1141

D S L L K I I N G N N Q P I L D T S S F

1141 AAG AAA CAA ATT AGT CAA ATC GAT AAA AAA ATC CAA AAG AAC TCA GAT TTG TAC TTG AAC 1201

K K Q I S Q I D K K I Q K N S D L Y L N

1201 GAT TTC ATT ACT ATG GAT GAA CTT AAG GAT AGA ACA GAT AGC CTG CAA GCA GAA AAA AAG 1261

D F I T M D E L K D R T D S L Q A E K K

1261 CTG CTC AAG GCC AAA ATC AGT GAA AAC AAA TTC AAC GAT TCC ACG GAT GTC TTT GAA CTC 1321

L L K A K I S E N K F N D S T D V F E L

1321 GTG AAA ACC CAA CTG GGT AGC ATC CCC ATC AAC GAA CTC AGC TAT GAT AAC AAA AAA AAG 1381

V K T Q L G S I P I N E L S Y D N K K K

1381 ATC GTT AAC AAC TTG GTT AGT AAG GTC GAT GTC ACC GCC GAT AAC GTC GAT ATC ATA TTC 1441

I V N N L V S K V D V T A D N V D I I F

1441 AAA TTC CAA CTG GCC TGA 1458

K F Q L A *
