# Supplementary material for: A Method for Producing Transgenic Cells Using a Multi-Integrase System on a Human Artificial Chromosome Vector
Source: PLoS One. 2011 Feb 24;6(2):e17267. doi: 10.1371/journal.pone.0017267 (PMC3044732; doi:10.1371/journal.pone.0017267)
Supplement: Figure S4 — Nucleotide sequence of mammalian codon-optimized Bxb1 integrase. The nucleotide sequence of the Bxb1 integrase used in this study. A mammalian codon-optimized Bxb1 integrase gene was synthesized de novo according to the native Bxb1 integrase amino acid sequence. (DOC) [file pone.0017267.s004.doc]

1 ATG AGG GCC CTT GTA GTT ATC AGG TTG AGT AGG GTT ACG GAT GCA ACC ACC AGC CCG GAG 61

M R A L V V I R L S R V T D A T T S P E

61 CGC CAA CTG GAA TCA TGT CAG CAG CTT TGT GCG CAG CGC GGC TGG GAC GTG GTG GGA GTG 121

R Q L E S C Q Q L C A Q R G W D V V G V

121 GCG GAG GAC TTG GAC GTT AGC GGG GCC GTT GAC CCA TTT GAC CGA AAG CGG AGA CCT AAC 181

A E D L D V S G A V D P F D R K R R P N

181 CTG GCT CGA TGG CTC GCC TTT GAG GAG CAG CCC TTC GAT GTG ATC GTC GCA TAC AGG GTC 241

L A R W L A F E E Q P F D V I V A Y R V

241 GAC AGA CTG ACC AGA TCC ATT CGC CAT CTG CAG CAG CTC GTT CAC TGG GCG GAG GAC CAC 301

D R L T R S I R H L Q Q L V H W A E D H

301 AAA AAG CTC GTG GTG AGT GCA ACA GAA GCC CAC TTT GAC ACC ACA ACA CCC TTC GCA GCC 361

K K L V V S A T E A H F D T T T P F A A

361 GTC GTG ATC GCT CTG ATG GGT ACC GTT GCC CAG ATG GAA TTG GAG GCA ATC AAG GAG CGG 421

V V I A L M G T V A Q M E L E A I K E R

421 AAC AGA TCC GCC GCT CAT TTC AAT ATC CGC GCG GGC AAG TAC AGG GGT AGT CTC CCA CCC 481

N R S A A H F N I R A G K Y R G S L P P

481 TGG GGG TAT TTG CCT ACC CGG GTG GAC GGC GAA TGG AGG CTT GTT CCC GAT CCC GTG CAG 541

W G Y L P T R V D G E W R L V P D P V Q

541 CGA GAG CGA ATA CTG GAA GTT TAT CAT CGA GTC GTG GAT AAC CAT GAA CCA CTC CAC CTG 601

R E R I L E V Y H R V V D N H E P L H L

601 GTG GCC CAC GAC CTT AAC CGA CGC GGC GTG CTG AGC CCT AAG GAC TAT TTT GCT CAA CTT 661

V A H D L N R R G V L S P K D Y F A Q L

661 CAG GGA AGA GAG CCA CAG GGT AGG GAA TGG TCA GCC ACA GCT CTC AAG CGG TCT ATG ATT 721

Q G R E P Q G R E W S A T A L K R S M I

721 TCC GAA GCA ATG CTC GGG TAC GCA ACA CTC AAT GGC AAG ACA GTT CGA GAC GAC GAC GGG 781

S E A M L G Y A T L N G K T V R D D D G

781 GCC CCC CTG GTT CGG GCC GAA CCC ATA CTT ACC CGC GAA CAA CTG GAG GCA CTT CGC GCG 841

A P L V R A E P I L T R E Q L E A L R A

841 GAA CTT GTG AAA ACA AGC CGA GCC AAA CCC GCA GTG AGC ACC CCA TCA CTG CTG CTG AGG 901

E L V K T S R A K P A V S T P S L L L R

901 GTG CTC TTC TGT GCC GTG TGC GGC GAA CCA GCA TAC AAG TTC GCT GGC GGG GGT CGA AAA 961

V L F C A V C G E P A Y K F A G G G R K

961 CAC CCC CGC TAC CGG TGT CGC TCA ATG GGT TTT CCA AAG CAC TGT GGC AAC GGA ACA GTT 1021

H P R Y R C R S M G F P K H C G N G T V

1021 GCA ATG GCC GAA TGG GAC GCT TTT TGT GAA GAA CAA GTG CTG GAT CTT CTG GGC GAC GCT 1081

A M A E W D A F C E E Q V L D L L G D A

1081 GAG AGG CTG GAA AAA GTA TGG GTG GCC GGG AGC GAC AGC GCC GTT GAG CTC GCC GAG GTG 1141

E R L E K V W V A G S D S A V E L A E V

1141 AAC GCC GAA TTG GTG GAC CTG ACG AGT CTC ATC GGA TCT CCA GCA TAC CGA GCT GGA TCC 1201

N A E L V D L T S L I G S P A Y R A G S

1201 CCC CAG CGA GAG GCT CTG GAC GCT CGG ATA GCC GCC CTG GCA GCA AGG CAG GAG GAG CTT 1261

P Q R E A L D A R I A A L A A R Q E E L

1261 GAG GGG TTG GAA GCA CGG CCT TCA GGA TGG GAA TGG CGG GAA ACA GGA CAG AGA TTT GGA 1321

E G L E A R P S G W E W R E T G Q R F G

1321 GAC TGG TGG AGG GAA CAG GAT ACC GCT GCT AAG AAC ACT TGG CTC AGG TCC ATG AAT GTT 1381

D W W R E Q D T A A K N T W L R S M N V

1381 CGA CTC ACC TTC GAC GTG AGG GGT GGG TTG ACC CGC ACC ATT GAT TTC GGG GAT CTG CAG 1441

R L T F D V R G G L T R T I D F G D L Q

1441 GAG TAT GAA CAG CAT CTC CGG CTT GGC TCC GTG GTA GAA AGA CTT CAT ACA GGC ATG TCA 1501

E Y E Q H L R L G S V V E R L H T G M S

1501 TGA 1503

*
